# Current Mathematical Models for Analyzing Anti-Malarial Antibody Data with an Eye to Malaria Elimination and Eradication

**DOI:** 10.1155/2015/738030

**Published:** 2015-12-06

**Authors:** Nuno Sepúlveda, Gillian Stresman, Michael T. White, Chris J. Drakeley

**Affiliations:** ^1^London School of Hygiene and Tropical Medicine, Keppel Street, London WC1E 7HT, UK; ^2^Centro de Estatística da Universidade de Lisboa, Faculdade de Ciências, Universidade de Lisboa, Bloco C6, Piso 4, Campo Grande, 1749-016 Lisboa, Portugal; ^3^MRC Centre for Outbreak Analysis and Modelling, Department of Infectious Disease Epidemiology, Imperial College London, Medical School Building, Norfolk Place, London W2 1PG, UK; ^4^Division of Population Health and Immunity, Walter and Eliza Hall Institute, 1G Royal Parade, Parkville, VIC 3052, Australia; ^5^Department of Medical Biology, The University of Melbourne, Parkville, VIC 3010, Australia

## Abstract

The last decade has witnessed a steady reduction of the malaria burden worldwide. With various countries targeting disease elimination in the near future, the popular parasite infection or entomological inoculation rates are becoming less and less informative of the underlying malaria burden due to a reduced number of infected individuals or mosquitoes at the time of sampling. To overcome such problem, alternative measures based on antibodies against specific malaria antigens have gained recent interest in malaria epidemiology due to the possibility of estimating past disease exposure in absence of infected individuals. This paper aims then to review current mathematical models and corresponding statistical approaches used in antibody data analysis. The application of these models is illustrated with three data sets from Equatorial Guinea, Brazilian Amazonia region, and western Kenyan highlands. A brief discussion is also carried out on the future challenges of using these models in the context of malaria elimination.

## 1. Introduction

Malaria is a global health problem with more than 1 billion people estimated to be at risk. This infectious disease is caused by* Plasmodium* parasites transmitted to humans through bites of infected Anopheles mosquitos. Geographically,* Plasmodium falciparum* (*P. falciparum*) parasites predominate in sub-Saharan Africa while* Plasmodium vivax* (*P. vivax*) is the major infectious agent in South America and Southeast Asia. According to the latest World Malaria Report [1], disease mortality and risk have been steadily decreasing in the last decade to the point that many countries are already targeting malaria elimination and eradication [2–5]. This decreasing trend in malaria transmission intensity, although highly beneficial to the affected populations, brings additional challenges to disease surveillance and elimination (reviewed in [6]). One of these challenges is related to the use of the current metrics of malaria risk in populations where disease transmission intensity is low and potentially affected by seasonal effects. The popular parasite rate is determined by the proportion of infected individuals at time of the survey. However, in low transmission settings, this measure is critically affected by the different performance of current diagnostic tools to detect the presence of infection while screening asymptomatic individuals. Another difficulty of using such measure is the high chance of finding only a few infected individuals in the sample, thus, having limited power to discriminate disease hotspots from other less-affected sites, as demonstrated in studies from Brazil [7] or Somalia [8]. The entomological inoculation rate is yet another popular measure of malaria risk. It is defined by the frequency at which people are bitten by infectious mosquitoes, thus, being informative on the direct interaction between the human and mosquito populations. The gold standard to estimate this measure is to use human-landing catches where mosquitoes are caught as they attempt to land on the exposed limbs of field workers [9, 10]. Although alternative methods exist in the literature, the estimation of the entomological inoculation rate is in general a laborious and time-consuming task in low transmission settings owing to a low number of infected mosquitoes [11]. It is also affected by seasonal effects and mosquito population dynamics and the degree of mosquito attractiveness to the human hosts or the chemicals used in the study [11].

To tackle the limitations of the above malaria risk measures, alternative indicators based on antibodies against different malaria antigens have been proposed [12] and tested in different epidemiological contexts [7, 8, 13–16]. The rationale of using antibody data is that the antibody concentrations in the serum are a direct correlate of parasite exposure, thus, providing information on current and recent infections. The temporal stability in antibody concentrations is an important advantage to reduce any seasonal effect on malaria transmission. In seroepidemiological studies, the most popular antibodies are those against the blood-stage apical membrane antigen-1 (AMA1) and merozoite surface protein-1 (MSP1) [7, 8, 13–16] owing to their broad immunogenicity and putative role in malaria vaccine development [17, 18]. Recent research identified other parasite targets [19, 20] but these remain to be tested in different epidemiological settings. Experimentally, antibody quantification is usually done by means of traditional enzyme linked immunosorbent assays [21]. Optical densities or titres in arbitrary units are then used for the subsequent data analysis. The most popular approach is to first define the serological status, seropositive or seronegative, of each individual. One then calculates the so-called seroprevalence that is defined by the proportion of seropositive individuals in the sample. Several studies showed an increased resolution of seroprevalence in discriminating sites with different endemicity levels in relation to parasite rate [7, 8]. Further analysis is then carried out in order to estimate current malaria transmission intensity. Since seroprevalence tends to increase with age as a result of augmenting immunity against malaria parasites, different stochastic models can be constructed for the data using age as a proxy of time. The common assumption to all these models is that individuals transit between seronegativity and seropositivity states upon malaria exposure or absence of it. In this scenario, one typically estimates the rate by which seronegative individuals become seropositive, the so-called seroconversion rate (SCR). SCR was found to correlate well with the parasite rate [13] or the entomological inoculation rate [12], thus, capturing the underlying malaria transmission intensity. Moreover, SCR also strongly correlates to the annual parasite index (the number of confirmed cases during 1 year/population under surveillance) × 1000—usually calculated by official health authorities [7].

This paper aims to review the mathematical and statistical aspects underlying the analysis of antibody data for inferring malaria transmission intensity. Special attention will be given to current methods aiming to define seropositivity and the subsequent mathematical models for estimating SCR under different epidemiological settings: stable malaria transmission intensity, abrupt reduction in SCR due to a malaria control intervention, change in SCR due to a putative age-dependent behavior, detection of migration effects, and detection of individual level heterogeneity through a set of covariates. Models for antibody acquisition using antibody titres themselves will also be described. Three different data sets from Bioko Island in Equatorial Guinea [15], Jacarecanga from the Brazilian Amazonia region [7], and western highlands from Kenya [22] are used to illustrate the application of these models to real-world problems. Finally, future analytical challenges will be discussed in the context of malaria elimination and eradication.

## 2. Mathematical Approaches to Analyzing Serology Data

### 2.1. Defining Seropositivity

In practice, there are two popular approaches to determine the serological status of an individual. The first approach uses an additional sample of nonexposed individuals in order to determine the distribution of the antibody levels referring to the underlying seronegative population. Statistically, the antibody levels of this sample are usually log transformed in order to approximately obtain a Gaussian distribution for the data. The serological classification of each individual in the sample is done by the 3*σ* rule for Gaussian distributions described in any introductory textbook of statistics. In more detail, this rule defines the range of antibody levels containing a 0.999 probability under the assumption of a Gaussian random variable for the data. One then classifies the individuals as seropositive if the respective antibody levels exceed the mean plus 3 times the standard deviation of the seronegative population, otherwise the individuals are considered as seronegative. This simple approach, despite ensuring a high probability of correctly classifying exposed individuals, has the disadvantage of underestimating seroprevalence.

The second approach focuses on the data under analysis only. The basic assumption is that the sample is composed of a mixture of latent seronegative or seropositive populations. The respective data is then analyzed by the so-called two-component mixture Gaussian model invoking a Gaussian distribution with average value *μ*_0_ and standard deviation *σ*_0_ for the seronegative population and another one with average value *μ*_1_ and standard deviation *σ*_1_ for the seropositive population. For independent and identically distributed random sample of *n* individuals, the corresponding sampling distribution is described by the following equation:

(1) fxi ∣ μ0,μ1,σ0,σ1,π=∏i=1n1−πfNμ0,σ0xi+πfNμ1,σ1xi,

where *x*_*i*_ is the antibody level of the *i*th individual in the sample, *f*_*N*(*μ*_0_,*σ*_0_)_(*x*_*i*_) and *f*_*N*(*μ*_1_,*σ*_1_)_(*x*_*i*_) are probability density functions of the Gaussian distributions associated with seronegative and seropositive populations, respectively, and *π* is the probability of sampling a seropositive individual from the population. Maximum likelihood estimation is facilitated by using the expectation-maximization (EM) algorithm that can be found in the mixtools package for the R software [23]. The next stage of the analysis is to assign each individual to each corresponding serological population. Again, one can use the 3*σ* rule as described above [14]. An alternative way to perform such classification is to jointly use the probabilities of classifying an individual with antibody level *x* as either seropositive or seronegative and then specify appropriate cut-off values to determine the serological status of each individual. The probabilities of classifying an individual with antibody level *x* as seropositive and seronegative are, respectively, given by

(2) p+ ∣ x=πfNμ1,σ1x1−πfNμ0,σ0x+πfNμ1,σ1x,p− ∣ x=1−p+ ∣ x.

The classification rule of the *i*th individual in the sample is then described as follows:

(3) $$\begin{array}{c} C_{i}=\left\{\begin{array}{cc} seronegative, & \text{if }x_{i}\leq c^{-}\\ indeterminate, & \text{if }c^{-}<x_{i}<c^{+}\\ seropositive, & \text{if }x_{i}\geq c^{+}, \end{array}\right. \end{array}$$

where *c* ^−^ and *c* ^+^ are the cut-off values in the antibody distribution that ensure a given classification probability, for instance, 90%. Note that individuals with antibody levels between *c* ^−^ and *c* ^+^ are deemed indeterminate due to the uncertainty in the corresponding serological classification. Besides checking whether model assumptions hold true on the data under analysis, an additional assessment of the quality of the classification rule is to report the size of this indeterminate region and the proportion of indeterminate individuals in the sample.

**Figure 1:**
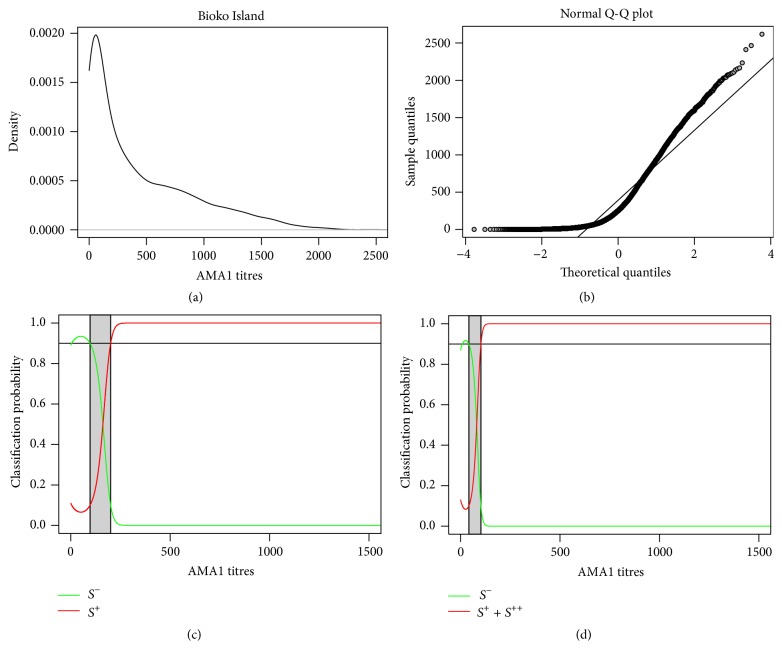
Determining seropositivity of anti-AMA1 antibodies from Bioko Island. (a) Probability density plot for the titre data. (b) Gaussian (or Normal) quantile-quantile plot for the data. (c) Classification probability curves predicted by the two-component Gaussian mixture model. (d) Classification probability curves predicted by the best three-component Gaussian mixture model where the intermediate component refers to a seropositive population.

*Example I* (Bioko Island). In 2004 the health authority of Equatorial Guinea launched integrated treatment and mosquito control programs in the Bioko Island. After 4 years of their initiation, a large cross-sectional survey was conducted at 18 sentinel sites in the island in order to assess the impact of these programs on malaria transmission [15]. IgG antibody levels of 6400 individuals were measured for* P. falciparum* AMA1 by ELISA. The antibody levels as measured by arbitrary titres range from −116.3 to 2618.9, suggesting a wide breadth of immune responses to this malaria antigen (Figure 1(a)). The average antibody level was 390.8 while the standard deviation was estimated at 457.4. As expected from data of a malaria endemic region, the corresponding quantile-quantile plot showed a strong departure of the data in relation to the Gaussian distribution due to presence of recently or currently exposed individuals with high antibody levels (Figure 1(b)). By fitting the above two-component Gaussian mixture model to the data, the serological status of each individual was determined by (3) with *c* ^−^ = 97.0 and *c* ^+^ = 200.8 (Figure 1(c)). These cut-off values suggested that 31.2% and 56.1% of the sample consisted of seronegative and seropositive individuals, respectively. The remaining 12.7% of the sample had unclear serological classification (Table 1). 

The above Gaussian mixture model can be extended to the setting where there are more than two components. Immunologically, such extension is in line with the notion that antibody levels can be boosted by frequent malaria exposure [24]. In this scenario, each component can be interpreted as corresponding to a specific degree of malaria exposure: not exposed, once exposed, twice exposed, three times exposed, and so forth.

Under the assumption of a known number of components for the data (say *K* + 1), the corresponding sampling distribution is given by

(4) $$\begin{array}{c} f\left(\;\left\{\;x_{i}\;\right\}\;|\;\left\{\;\mu_{k},\sigma_{k},\pi_{k}\;\right\}\;\right)=\overset{n}{\underset{i=1}{\prod }}\left[\;\overset{K}{\underset{k=0}{\sum }}\pi_{k}f_{N\left(\mu_{k},\sigma_{k}\right)}\left(\;x_{i}\;\right)\;\right], \end{array}$$

where *μ*_0_ < *μ*_1_ < *μ*_2_ < ⋯<*μ*_*K*_ are the averages of the population not exposed, once exposed, twice exposed,…, and *K* times exposed, respectively, *σ*_0_, *σ*_1_, *σ*_2_,…, *σ*_*K*_ are the corresponding standard deviations, and *π*_0_, *π*_1_, *π*_2_,…, *π*_*K*_ are the corresponding sampling probabilities (with *π*_0_ = 1 − ∑_*k*=1_^*K*^). The conditional classification probabilities of seropositive and seronegative individuals given antibody level *x* can be generalized as follows:

(5) p+ ∣ x=∑k=1KπkfNμk,σkx∑k=0KπkfNμk,σkxi,p− ∣ x=1−p+ ∣ x.

The corresponding classification rule is also given by (3) but now the cut-off values must be recalculated according to these new classification probabilities. As for the two-component Gaussian mixture model, maximum likelihood estimation via EM algorithm can also be performed to estimate the unknown parameters {*μ*_*k*_, *σ*_*k*_, *π*_*k*_, *k* = 0,…, *K*}. Starting this estimation algorithm with different initial conditions is recommended to obtain the correct convergence to the global maxima of the log-likelihood function.

An important question in practice is to know how many components one must consider to describe the data well. In terms of maximum likelihood estimation, this question can be answered by using the profile likelihood method. This method proceeds as follows: (i) start the analysis with *K* = 1, (ii) obtain the corresponding maximum likelihood estimates and then calculate the respective value of the likelihood function, (iii) add another component into the analysis and repeat step (ii), and (iv) keep increasing the number of components until reaching a realistic maximum value for that parameter. The optimal number of components is the one providing the maximum value of all maximum likelihood values calculated for each number of components considered in the analysis. The profile likelihood method, despite estimating the total number of components, brings potential problems of model overfitting and uncertainty in the classification rule. Overfitting can be obtained by considering a model with too many components. This problem can be surpassed by using different information measures with the aim of weighting the quality of the data fitting with the intrinsic complexity of a model. The most popular information measure is the Akaike's information criterion (AIC) defined by twice the absolute value of the log-likelihood function evaluated at the maximum likelihood estimates (measuring the quality of the respective data fitting) plus twice the total number of estimated parameters (estimating the intrinsic model complexity). Since models are penalised in this criterion as function of the total number of parameters, one should choose the model that shows the lowest AIC value among all models tested. Uncertainty in the classification rule can arise from data where the different serological populations are tight together in the antibody distribution. A simple solution is to choose the model with the highest likelihood but implying a sufficiently clear serological classification of the individuals in the sample.

An additional difficulty in using a Gaussian mixture model with more than two components is the ambiguity in linking each component to the corresponding serological status. Let us consider the three-component mixture model for the moment. In this setting, the components with the lowest and highest average titres are easily interpreted as related to seronegative and seropositive populations, respectively. On the one hand, the component with intermediate average titres can be interpreted as a seronegative population if one assumes two populations with different genetic backgrounds. This interpretation agrees with studies from Burkina Faso where the Fulani typically have higher antibody concentrations at baseline in comparison to other ethnic groups living in the same area [25, 26]. On the other hand, this intermediate component can also be interpreted as a seropositive population under the assumption of immunity boosting upon recurrent malaria exposure as described above. This and the component with the highest average concentrations are then related to exposed and boosted populations, respectively. Similar reasoning can easily be applied to the scenario of a higher number of components. For that one just needs to consider the putative existence of more than one seronegative and seropositive population. In absence of additional information about the populations under study, it is difficult to resolve the ambiguity about component interpretation. A possible solution is to first understand how the performance of the classification is affected by changing the interpretation of the components and then make a judgement call upon the reasonability of the corresponding results. 

*Example I* (Bioko Island continued). Previous analysis was extended to fit Gaussian mixture models with more than two components. Models with three and four components seemed to describe the data better than the one with two components only, according to AIC (Table 1). Despite providing a good balance between data fitting and model complexity, the four-component models implied high percentages of individuals with unclear classification (>22%). The best model would appear to be the one with three components where the second and the third components were interpreted as referring to seropositive populations. This model improves the quality of the data fitting and implied a percentage of individuals with unclear classification (14%) similar to the one obtained from the two-component model. Comparing to previous results for the two-component model, the inclusion of a third additional component suggested that the seropositive population could in fact be split into exposed and boosted individuals with average antibody titres of 214.0 and 848.3, respectively. The new cut-off values for the classification rule led to the classification of 19.3% and 66.8% of the sampled individuals as seronegative and seropositive, respectively. 

Recent research has highlighted the great potential of using Bayesian approaches in Gaussian mixture models. The major advantage of these methods is to provide a coherent and elegant analytical framework for estimating the total number of components from the data. Since this number is unknown quantity, it is considered as random variable with a given probability distribution before conducting data analysis the so-called prior distribution. Bayes theorem then allows linking the prior distributions for all unknown parameters with the sampling distribution of the data. As a result, prior distributions of the parameters are updated by the data, giving rise to the so-called posterior distributions. These latter distributions are then the core of the Bayesian statistical inference. The current success of Bayesian approaches is intimately related to the use of powerful simulation methods in order to determine the posterior distributions given the data. In Gaussian mixture models, the Markov Chain Monte Carlo with reversible jumps is a popular choice for posterior estimation [27]. Similar simulation algorithm can theoretically be applied to multivariate Gaussian mixture models [28]. These models are particularly suitable for analyzing data of more than one malaria antigen simultaneously (e.g., for analyzing AMA1 and MSP1 data together). A Bayesian two-component mixture model using arbitrary probability distributions for the latent populations was proposed for classifying fever and nonfever malaria cases according to the underlying parasitaemia [29, 30]. Up to now a single seroepidemiological study in malaria [14] is known to have analyzed data via Bayesian methods and, thus, little can be said about their performance in practice.

### 2.2. Detecting Stable Malaria Transmission Intensity Using Seropositivity Data

**Figure 2:**
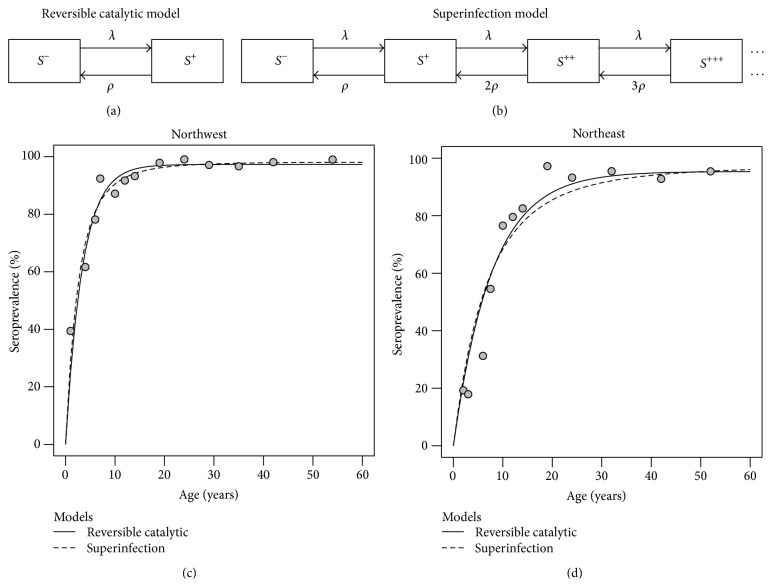
Analysis of seropositivity data. (a) Compartmental representation of the reversible catalytic model where individuals transit between seronegative and seropositive states with rates *λ* (SCR) and *ρ* (SRR). (b) Compartmental representation of the superinfection model in which there are multiple seropositive states owing to immunity boosting upon recurrent malaria infections. (c) Analysis of seropositivity AMA1 data from northwest region of Bioko Island under the assumption of stable malaria transmission over time. (d) Similar data analysis for northeast region of Bioko Island. In plots (c) and (d), the dots represent the observed seroprevalence of distinct age groups by splitting the sampled age distribution into 7.5% centiles. To plot each seroprevalence, the median value of each age group was used.

After classifying individuals into their serological status, the corresponding data analysis proceeds by estimating stochastic models that aim to inform about the underlying malaria transmission intensity. The most popular models belong to the class of the reversible catalytic models (RCMs) [31–33]. When applied to serological data from infectious diseases that do not induce long-lasting immunity, such as the case of malaria, these models assume that age is deemed an appropriate proxy of the historical time so that data from each individual can be seen as a random realization of a seroconversion-seroreversion stochastic law. More precisely, individuals are born as seronegative but can be converted into seropositive upon malaria exposure. In the absence of frequent malaria exposure, individuals can revert to a seronegative state (Figure 2(a)). Mathematically, this idea can be described as a Markov chain model where one must specify the average rates by which the individuals become seropositive and return to the seronegative, the seroconversion, and seroreversion rates (SCRs and SRRs), respectively. Epidemiologically, SCR is related to the underlying disease transmission intensity as it correlates well with typical malariometrics, such as parasite rate or entomological inoculation rate. It is also related to (host) factors affecting antibody production. In contrast, SRR reflects host factors (e.g., genetics or age) affecting antibody decay in absence of malaria infection.

The simplest model for the data is to assume stable and constant malaria transmission intensity over time. A fixed SRR is also assumed because seropositivity data has limited power to describe variations in that parameter. For mathematical simplicity, the seroconversion-seroreversion dynamics of each individual is easily described by a Markov chain with two states, seronegative (*S* ^−^) and seropositive (*S* ^+^). The resulting RCM is described by the following probability of an individual aged *t* being seropositive:

(6) $$\begin{array}{c} p_{S^{+}}\left(\;t\;\right)=\frac{\lambda }{\lambda +\rho }\left(\;1-e^{-\left(\lambda +\rho \right)t}\;\right), \end{array}$$

where *λ* and *ρ* are the SCR and SRR, respectively. It is worth noting that the above probability is an increasing function of age reaching a plateau at *λ*/(*λ* + *ρ*) when age goes to infinite.

The above model can be extended to the so-called superinfection model (SIM), where immunity boosting can occur owing to recurrent malaria infections [24]. In line with the Gaussian mixture models with more than 2 components for antibody titre data, the notion of boosting can be translated into distinct seropositive states, for instance, *S* ^+^,  *S* ^++^, and *S* ^+++^, depending on the cumulative level of malaria exposure (Figure 2(b)). In particular, a seronegative individual becomes a first-order seropositive upon a malaria infection. This same individual while still being first-order seropositive can evolve to a second-order seropositive upon an additional malaria exposure and so forth. A practical implication of this idea is a longer sojourn time in the seropositive state(s) in relation to the one predicted by RCM. Moreover, since there are multiple latent seropositive states, the estimates of the seroconversion rate tend to be higher in this model than in its reversible catalytic counterpart for the same data. The probability of an individual aged *t* being at any seropositive state is now given by

(7) $$\begin{array}{c} p_{S^{*}}\left(\;t\;\right)=1-e^{-\left(\lambda /\rho \right)\left(1-e^{-\rho t}\right)}, \end{array}$$

where *S* ^*∗*^ represents the set of all possible seropositive states an individual can belong to. More details on the corresponding mathematical derivation can be found elsewhere [24]. In practice, the application of this model to real-world problems shows limitations in terms of estimation [34]. On the one hand, SIM and RCM are approximately equivalent to each other in low transmission settings due to the rarity of boosting events. On the other hand, seroreversion is a rare event in high transmission settings due to boosting. Thus, for the matter of simplicity, a model considering seroconversion only is more reasonable for that situation. Interestingly, (6) and (7) when *ρ* → 0 can be rewritten as the classical complementary log-log model [35]:

(8) $$\begin{array}{c} \log\left[\;-\log\left(\;1-p_{S^{+}}\left(\;t\;\right)\;\right)\;\right]=\log\lambda +\log t. \end{array}$$

Despite having limited application in malaria research [36], the complementary log-log model has been used in nonmalaria immunological settings where a single immunization is thought to exert a permanent seropositive phenotype [37, 38].

With respect to model estimation, seropositive data adjusted for age is organized as a two-way frequency table with *A* rows and two columns, where *A* is the total number of different age values in the sample and the two columns refer to the serological status of the individuals (i.e., seronegative and seropositive). In this data format, the sampling distribution is assumed to be a Binomial-product sampling distribution, an independent Binomial distribution per age value and probability of success given by the model under fitting; that is,

(9) fmt ∣ nt,λ,ρ=∏t=1Antmtπtmt1−πtnt−mt,

where *m*_*t*_ and *n*_*t*_ are the frequency of seropositive and all individuals aged *t* years, respectively, and *π*(*t*) is the expected seroprevalence at age *t* described by (6), (7), or (8) if estimating RCM, SIM, or the complementary log-log model, respectively. Maximum likelihood estimation can be applied to the data. Stata and R scripts for data fitting are currently available from the authors upon request. 

*Example I* (Bioko Island continued). As mentioned earlier, the cross-sectional survey from Bioko Island consisted of 18 sentinel sites spread over the island. To increase statistical power, the corresponding data was analyzed by considering 5 major geographical regions: northeast, northwest, southeast, southwest, and Malabo. A comprehensive analysis of this data set can be found in the original study report [15]. For illustrative purposes, the statistical analysis was carried out on data from northeast and northwest regions specifically. According to the seropositivity determination step, there are 1332 and 877 individuals with an assigned serological status from northwest and northeast regions, respectively. The corresponding overall seroprevalence was estimated at 86.7% (95% CI: 84.8%–88.5%) and 69.9% (95% CI: 66.7%–72.9%). These estimates are higher than the ones reported in the original study (69.2% and 46.6%, resp.) because this study used a two-component Gaussian mixture model for titre data, thus, predicting a higher cut-off value for seropositivity (Table 1). As expected from a malaria endemic area, the seroprevalence increased with age in both regions (Figures 2(c) and 2(d)). With respect to northwest region, both models described the seroprevalence curve well (Figure 2(c)). However, SIM provided a slightly better fit to the data than RCM (log-likelihood = −69.71 and −71.31, resp.), a result in line with the use of a three-component mixture model for seropositivity determination. Also in agreement with theoretical expectations was the higher SCR obtained from SIM in relation to the one predicted by RCM (0.359 versus 0.286; Table 2). For northeast region, the overall seroprevalence is decreased, thus, implying lower SCR estimates for RCM and SIM (0.124 and 0.139 for RCM and SIM, resp.). Although RCM showed a better fit to the data than its SIM counterpart (log-likelihood = −96.87 and −102.95, resp.), both models overestimated the seroprevalence of young aged individuals (up to 10 years ago) (Figure 2(d)) and, thus, they could not be considered as good candidate models for the data. Such overestimation suggested that young aged and older individuals have different serological dynamics that cannot easily be captured by a stable malaria transmission assumption. An easy explanation is the putative reduction in malaria transmission intensity after the initiation of malaria control programs in 2004 in the island. This and other related topics will be explored in the following section.

### 2.3. Detecting Heterogeneity in Malaria Transmission Intensity Using Seropositivity Data

A unique advantage of using serology data is the possibility of detecting heterogeneity in disease transmission across different epidemiological situations. This advantage has been demonstrated in several studies where seroprevalence taken as a function of age qualitatively changes at a given age value. Such change might be attributed to an abrupt reduction in malaria transmission after the initiation of a malaria control or elimination program [15, 16]. Similar phenomenon was found for Trachoma [39] or Chagas disease [40]. Another possible explanation for that change is related to distinct malaria risk between young and older individuals owing to behavioral factors [41]. A third and last explanation is the occurrence of migration waves over time [42], as observed in Chagas disease [43]. A detailed description of these scenarios follows.

#### 2.3.1. Detecting Historical Changes in Malaria Transmission

The commitment of many national health authorities in reducing or targeting elimination in future years brings future challenges in assessing the real impact of the designed interventions on the target populations. This assessment can be made by analyzing seropositivity data conveniently. For that one assumes there was an abrupt reduction of malaria transmission intensity at some time point before data collection. It is expected that an abrupt reduction in malaria transmission intensity would translate in a similar effect on the SCR. Sampled individuals are then split according to their date of birth in relation to the calendar time when the reduction in malaria transmission intensity actually occurred. More precisely, the serological history of individuals born before that reduction contemplates a first time period where the past SCR operates followed by a second period where the current SCR sets the rules. In contrast, individuals born after the reduction would lie down on that second time period and, thus, their serological dynamics are simply described by previous RCM and SIM for stable malaria transmission.

To calculate the seroprevalence of an individual with age *t* that experienced a reduction in malaria transmission intensity at time *τ* before sample collection, one must consider the sum of two probabilities associated with the following mutually exclusive events: (i) an individual became seropositive between birth and *t* − *τ* and remained so after that and (ii) an individual remained seronegative between birth and *t* − *τ* and became seropositive after that. Since RCM can be formulated as a two-state Markov chain, the seroprevalence for individuals with age *t* is calculated by multiplying the vector of probabilities associated with an individual being seropositive and seronegative at time *t* − *τ* (see (6)) by the probability transition matrix of the second Markov chain associated with the current SCR and evaluated at time *τ*. The resulting expected seroprevalence is then given by

(10) pS+t=θ21−e−γ2τ+θ11−e−γ1t−τe−γ2τ,if  t>τθ21−e−γ2t,if  t≤τ,

where *θ*_*i*_ = *λ*_*i*_/(*λ*_*i*_ + *ρ*), *γ*_*i*_ = *λ*_*i*_ + *ρ*, *i* = 1, 2, *λ*_1_ and *λ*_2_ are the past and current SCR under the restriction of *λ*_2_ < *λ*. Similar argument can be applied to the superinfection model, leading to the following seroprevalence:

(11) pS+t=1−e−λ1/ρe−ρτ−e−ρt−λ2/ρ1−e−ρτ,if  t>τ1−e−λ2/ρ1−e−ρt,if  t≤τ.

With respect to parameter estimation, the sampling distribution is again assumed to be a Binomial-product distribution ((9), where *π*(*t*) is described by (10) or (11)). To estimate all parameters (*λ*_1_, *λ*_2_, *ρ*, and *τ*) via maximum likelihood, a profile likelihood approach is usually applied to the data under analysis: (i) set *τ* = 1, (ii) determine the respective maximum likelihood estimates for the remaining parameters, (iii) calculate the corresponding log-likelihood function, (iv) increase one unit to *τ* and repeat steps (ii-iii), and (v) keep increasing *τ* until reaching the maximum expected value for that parameter. The overall maximum likelihood estimates are those associated with the value of *τ* that provides the maximum value of all log-likelihood values. Although statistically sound, this method tends to overestimate the true change point (i.e., estimates located further in past than they should), even if using a large sample size (our own results). This suggests that seropositivity data might not have enough information to estimate that parameter with high precision. Therefore, the interpretation of a specific estimate for the reduction time point should be done with caution. In practice, models assuming a stable or an abrupt reduction in malaria transmission intensity must compare to each other for the same data. A log-likelihood ratio test can then be applied to the corresponding results using the following test statistic under the null hypothesis:

(12) $$\begin{array}{c} L=\left(\;-2\;\right)\times \left(\;\Lambda_{stable}-\Lambda_{reduction}\;\right)⇝\chi_{\left(2\right)}^{2}, \end{array}$$

where Λ_stable_ and Λ_reduction_ are the log-likelihood functions evaluated at the maximum likelihood estimates for the models assuming a stable or an abrupt reduction in malaria transmission intensity, respectively, and *χ*_(2)_^2^ is a Chi-square distribution with the two degrees of freedom resulting from the difference in the total number of parameters of the models under testing (*λ* and *ρ* versus *λ*_1_, *λ*_2_, *ρ*, and *τ*). For a 5% significance level, *p* values < 0.05 show evidence for a significant change in disease transmission.

With the increasing complexity of the models under analysis, statistical inference via maximum likelihood methods becomes more cumbersome due to possible lack of convergence of the numerical algorithms leading to maximum likelihood estimates [15] and the inaccuracy of large sample approximations for the confidence intervals and test statistics [34]. These problems can be surpassed by using Bayesian inference. In this approach, each parameter in a model has an associated prior distribution that, in turn, is updated with the observed data by means of Bayes theorem. The resulting distribution is in the core of Bayesian inference and called posterior distribution. Posterior mean and median of this distribution are two possible Bayesian estimates for the same parameter. Credible intervals are the Bayesian equivalent to the confidence intervals of classical statistics and calculated by the appropriate quantiles of the posterior distribution that ensure a given probability mass (i.e., 95%). Model comparison can be performed via AIC or other Bayesian information measures, such as the Deviance Information Criterion (DIC) [44]. Theoretically, DIC is defined by the posterior mean of the deviance function (twice the absolute value of log-likelihood function) plus the effective number of parameters of a given model. In turn, the effective number of parameters is calculated by the difference between the posterior mean of the deviance function and the same function evaluated at the posterior means of the parameters. Likewise with AIC, one should choose the model that shows the lowest DIC value among all models tested.

In general, there are two major difficulties in performing Bayesian analysis. The first one relates to how to choose the prior distributions for the unknown parameters. One solution is to use noninformative prior distributions in situations where prior information about the parameters of interest is limited or scarce. Popular choices for noninformative prior distributions are the uniform distribution for parameters defining probabilities or Gaussian distributions with mean 0 and sufficiently large standard deviation for parameters defined in real space. In contrast, if one has strong prior beliefs about the parameters of interest, informative prior distributions can be elicited. Prior elicitation is generally based on a convenient probability distribution (e.g., a Gaussian distribution) upon which one determines the corresponding prior parameters—the so-called hyperparameters—by conjugating the expected prior mean with a set of prior quantiles set for that distribution. Although informative prior distributions are in line with the permanent dialogue between inductive and deductive reasoning intrinsic to the scientific method, most researchers adopt a conservative strategy to data analysis by using noninformative prior distributions for the unknown parameters. The second difficulty concerns the calculation of the posterior distributions. However, this is greatly reduced by the powerful Markov Chain Monte Carlo (MCMC) that, virtually, can deal with any kind of model complexity. In practice, R/Jags is an easy-to-use package for MCMC computing. Illustrative scripts for the above RCM and SIM are available from the authors upon request. 

**Figure 3:**
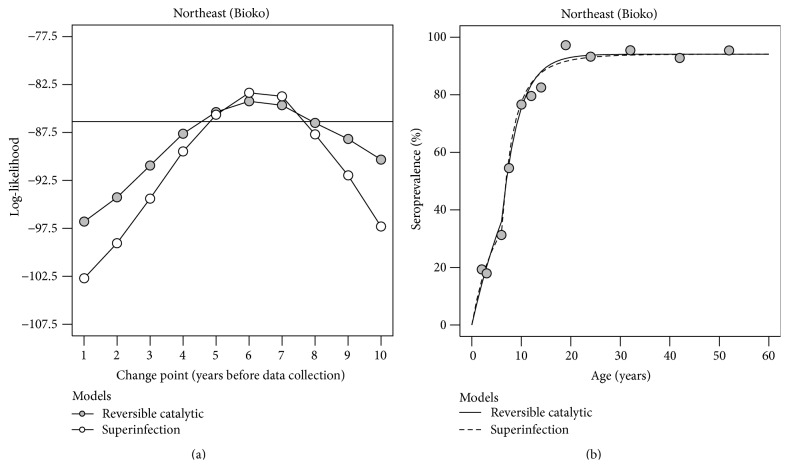
AMA1 seropositivity data analysis of northeast region from Bioko Island under the assumption of a past abrupt reduction in malaria transmission intensity. (a) Profile likelihood plot to estimate the best change point for the reversible catalytic model, where the solid and dashed lines refer to the log-likelihood value for the model assuming a stable transmission intensity and the cut-off value accepting that model at a 5% significance level, respectively. (b) Maximum likelihood fits of the reverse catalytic and superinfection models assuming an abrupt reduction in malaria transmission intensity estimated to have occurred 6 years before sampling.

*Example I* (Bioko Island continued). As highlighted earlier, the fits of RCM and SIM assuming stable malaria transmission intensity suggested a variation in malaria risk between younger and older individuals living in the northeast region of the island (Figure 2(d)). Such variation might be attributed to a reduction in malaria transmission intensity owing to a known malaria control initiative launched in 2004. To test this hypothesis, RCM and SIM with an abrupt reduction in malaria transmission intensity were fitted to the data via maximum likelihood estimation. The most likely reduction point for both models was 6 years before data collection (Figure 3(a)); the corresponding 95% confidence intervals were 4.2–8.4 and 4.8–7.7 for RCM and SIM, respectively. Both models were in close agreement with the data visually (Figure 3(b)) and better than the previous ones assuming stable transmission, according to likelihood ratio test (*p* values < 0.001). SIM led to a higher log-likelihood value than its RCM counterpart (Table 2) and, thus, it might be deemed the best model for the data. Again, this result is consistent with the choice of three-component Gaussian mixture model for the corresponding titre data. Previous and current SCRs were estimated at 0.900 and 0.098 for SIM and at 0.274 and 0.077 for RCM. These implied a reduction in malaria transmission intensity of around 89% and 72% for SIM and RCM, respectively. Note the putative overestimation of the time point for the reduction event (6 years before sampling versus the time when the Bioko malaria control initiative started). This result is in line with ongoing research where the profile likelihood method overestimated the true change point from simulated data typically found in African population (our own results).

#### 2.3.2. Detecting Changes in Malaria Exposure due to Age-Dependent Behaviors

A very similar age-adjusted seroprevalence curve to previous case can be found for populations where older individuals have a higher malaria transmission intensity compared to the one for younger individuals due to an age-dependent behavior factor. A typical example is the commute of adults to working sites that are malaria transmission hotspots in contrast to children and adolescents who do not travel to those sites. This situation was reported for some populations living in the forests of Cambodia and Indonesia [41, 45]. The above RCM and SIM are easily translated to this new situation. More precisely, both younger and older individuals share the same SCR until a certain age. Then SCR abruptly increases to a new level in a similar way as previous case. Therefore, (10) for an abrupt reduction in malaria transmission intensity can be adapted as follows:

(13) pS+t=θ11−e−γ1τ+θ21−e−γ2t−τe−γ1τ,if  t>τθ11−e−γ1t,if  t≤τ,

where *θ*_*i*_ = *λ*_*i*_/(*λ*_*i*_ + *ρ*), *γ*_*i*_ = *λ*_*i*_ + *ρ*, *i* = 1, 2, *λ*_1_ and *λ*_2_ are the SCR for younger and older individuals, respectively, under the restriction of *λ*_1_ < *λ*_2_. For the superinfection assumption, the resulting model can be expressed as follows:

(14) pS+t=1−e−λ2/ρe−ρτ−e−ρt−λ1/ρ1−e−ρτ,if  t>τ1−e−λ1/ρ1−e−ρt,if  t≤τ.

Parameter estimation and model comparison can be performed via maximum likelihood and Bayesian methods as described above for the models with an abrupt change in malaria transmission intensity. 

**Figure 4:**
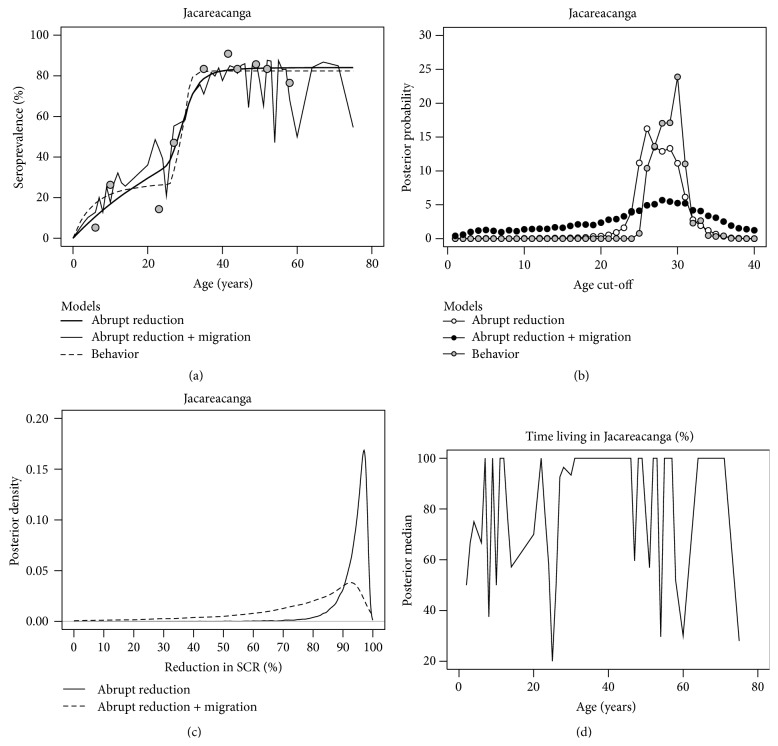
Analysis of* P. falciparum* seropositivity data from Jacareacanga (Brazil) using Bayesian methods. (a) Seroprevalence curves as predicted by RCMs assuming an abrupt reduction in SCR with and without migration and assuming a behavioral factor dependent on a given age cut-off, where dots represent the observed seroprevalence for age groups by splitting the age distribution in deciles. (b) Posterior distributions for the age cut-off for the models mentioned in (a). (c) Posterior probability densities for the reduction in SCR assuming or not migration effects. (d) Posterior median for the fraction of time living in the area in relation to the corresponding age of the individuals, as expected from RCM assuming migration effects and an abrupt reduction in SCR.

*Example II* (Jacareacanga, Brazil). A recent study was conducted in the Brazilian Amazonia region [7] where* P. vivax* is currently the major malaria threat opposed to what occurred in the past where* P. falciparum* infections predominated. A total of around 1300 individuals were sampled from 7 different municipalities in Pará state. Previous analysis suggested stable malaria transmission for* P. vivax* infections but detected a putative abrupt reduction of* P. falciparum* transmission intensity estimated to have occurred around 25–30 years before sampling. Although this change is in line with the intensification of malaria control initiatives by Brazilian health authorities in the area, alternative explanations were also discussed but not formally tested. More precisely, gold mining is one of the key economic activities in the area but also an important risk factor for malaria transmission. Mining was also a determinant factor of the known migration wave from nonendemic states to the region since 1970s. In this line of thought, the detection of a change occurred 25–30 years before sampling might be confounded by the increased malaria risk of the older population that are typically miners. This hypothesis is now tested against the one assuming an abrupt reduction of malaria transmission intensity. The analysis is focused on the* P. falciparum* seropositivity data from the municipality of Jacareacanga where the past reduction in SCR seemed more pronounced (i.e., from 0.514 to 0.017 [7]). Data under analysis comprised a total of 172 individuals of which 2.3% were infected with malaria parasites at the day of the survey. The seroprevalence for any* P. vivax* and* P. falciparum* antigens was estimated at 69.2% and 59.3%, respectively, using a two-component Gaussian mixture model for the corresponding titre data. These estimates suggested a high malaria endemicity for that municipality as issued by the Brazilian authority for malaria control but using the recorded annual parasite index. In contrast to previous example, Bayesian methods were alternatively applied to the data using the following noninformative prior distributions for the parameters: (i) Gaussian distributions with mean 0 and standard deviation 103 for all SCRs and SRRs in log scale and (ii) a discrete uniform distribution between 1 and 40 for the age cut-off. Since seropositivity data was previously derived from a two-component Gaussian mixture model, this analysis is based on the RCM only. MCMC simulation via R/Jags package was used to obtain the posterior estimates for the parameters; a long chain of 1,050,000 iterations was generated where the first 50,000 were discarded as the burn-in period and a lag of 100 was used to reduce correlation between simulated values. As previously reported, the model assuming an abrupt reduction in SCR captures data well (Figure 4(a)). However, there was some degree of uncertainty on the time in which that reduction had occurred (Figure 4(b)). The posterior mean and median were consistent with a sudden drop in* P. falciparum* transmission intensity 28 years before data collection (e.g., around 1980). The posterior mean for past and current SCR was 0.436 and 0.019, respectively, while the corresponding posterior medians were 0.386 and 0.019 (Table 2). In agreement with a Bayesian analysis using noninformative prior distribution, these posterior estimates implied a reduction in SCR in the same order of magnitude to that obtained in the original study. The model assuming a behavioral factor also fitted the data well (Figure 4(a)) with a slightly higher age cut-off for the occurrence of such behavior (around 29 years old). Again, there was some uncertainty associated with the age value where that behavior becomes epidemiologically relevant. In absence of that putative behavioral factor, the baseline SCR was estimated at 0.051 or 0.046 if one chooses the posterior mean or median, respectively (Table 2). This SCR increased to the posterior mean and median of 0.654 and 0.693 at older ages. DIC was then used to compare both models. The respective DIC estimates are 89.72 and 96.67 for the RCMs assuming an abrupt reduction and a change in SCR due to an age-dependent behavioral factor. Since the best model is the one that shows the lowest value for DIC, the change in SCR seemed to be better explained by an abrupt reduction in* P. falciparum* transmission intensity rather than the existence of a putative risk factor dependent on age, such as those related to gold mining activities in the heart of the Amazonia forest.

### 2.4. Detecting Migration Effects on Malaria Exposure

Up to now all models were analyzed under the assumption of stable populations with no migration effects. This assumption is reasonable in most studies because individuals living shortly in a given study area are typically excluded from the survey. However, in the current era of facilitated movement between populations, it might be difficult to recruit locally born individuals only, thus, affecting the estimation of the SCR. In one extreme, the easiest migration setting is the importation of malaria cases to nonendemic regions where there are no sufficient conditions for efficient malaria transmission. In this case, there is no strong rationale to use any of the above models since SCR would reflect the disease transmission intensity of the places where the sampled individuals come from. On the other extreme, migration from nonendemic region to endemic ones might introduce bias on SCR estimates. More precisely, at the time of migration, individuals are immunologically naive to malaria parasites in comparison to those with the same age but locally born in the region. This is the case of the history of malaria in Brazil where a gold rush in 1970s led to the migration of thousands of people from nonendemic states to the heart of the Amazonia forest [42]. Such migration caused an increase in population size and malaria cases in the region. Another known example from the literature is the founder effect in a Peruvian community affected by Chagas disease where locally born individuals and founders have distinct seroprevalence histories [43].

Until now age was considered a proxy of the total exposure time of each individual to malaria antigens. In the situation where individuals migrated from a nonendemic region to an endemic one, age used in all above models is simply replaced with the total residence time of each individual in the endemic area if available. In practice, such information is not routinely collected, thus, requiring additional estimation. Without lack of generality, let us focus on the simple RCM with stable malaria transmission intensity. Similar argument can be applied to the remaining models. As seen earlier, the expected seroprevalence curve is given by (6). The same model including migration effects is described as follows:

(15) $$\begin{array}{c} p_{S^{+}}\left(\;t\;\right)=\frac{\lambda }{\lambda +\rho }\left(\;1-e^{-\left(\lambda +\rho \right)t^{*}}\;\right), \end{array}$$

where *t* and *t* ^*∗*^ are the age and the total residence time of an individual living in an endemic area, respectively. In absence of information of the total residence time, the estimation of *t* ^*∗*^ can be done by considering *t* ^*∗*^ = *t* × *p*_*t*_, where *p*_*t*_ ∈ (0,1] is the proportion of residence time of individuals with age *t*. In practice, estimation of each *p*_*t*_ might be cumbersome by maximum likelihood methods. Firstly, the above RCM and SIM are intrinsically nonlinear and these additional unknown parameters might lead to convergence problems of the respective maximization algorithms. Secondly, sample information might be insufficient to provide accurate estimation of the residence time of each individual. Alternatively, Bayesian methods can overcome some of these limitations. As mentioned earlier, Bayesian inference is nowadays facilitated by the availability of powerful MCMC simulators that can estimate any kind of statistical model. Moreover, Bayesian inference can also coherently integrate external information on the residence time by describing the prior distribution accordingly. In so doing, one can consider the following family of prior distributions for *p*_*t*_:

(16) Ppt=x=p0,if  x=1,1−p0xαt−11−xβt−1Beαt,βt,if  0<x<1,

where *p*_0_ is the prior probability of an individual with age *t* being locally born and *p*_*t*_ for a migrant is modeled a priori by a Beta distribution with hyperparameters *α*_*t*_ and *β*_*t*_. If little information is known about the migrant population, one can specify *α*_*t*_ = 1 and *β*_*t*_ = 1 in order to obtain uniform distribution. Note that the parameter *p*_*t*_ is a priori allowed to vary with age. This is particularly useful to capture migration effects of specific age groups, such as adults who tend to migrate for work reasons. 

*Example II* (Jacareacanga, Brazil, continued). As mentioned above, the history of malaria in Brazil is intimately related to a gold rush in 1970s from nonendemic regions to endemic ones in the heart of the Amazonia forest [42]. Since mining is the main economic activity of Jacarecanga, it is possible that the above past and current SCR estimates can be improved in order to take into account any past migration effects. Unfortunately data concerning time of residence were not consistently recorded across individuals and study sites in the original study and, thus, the parameters *p*_*t*_s were directly estimated from seropositivity data. The above data analysis was then extended to the situation of RCM assuming an abrupt reduction in the disease transmission intensity together with putative migration effects described by (10). Little information was known about the migrant population and, thus, a prior uniform distribution for the parameters *p*_*t*_'s was specified for the Bayesian analysis. With respect to the prior probability *p*_0_, the Brazilian Office for Geography and Statistics states that 14.4% of the population living in Jacareacanga in the 2010 census were not born in the north states comprising the Amazonia region [46]. Therefore, it seemed unlikely that the percentage of the migrant population from Jacareacanga was lower than 15%. To understand the impact of *p*_0_ on the subsequent inferences, different values for that hyperparameter were tested, specifically, 0.25, 0.50, 0.75, and 0.9. The best one appeared to be 0.75 because it implied the highest posterior median and mean of the log-likelihood function (results not shown). The introduction of migration effects in the RCM with an abrupt reduction resulted in a seroprevalence curve with a more complex pattern (Figure 4(a)). However, this higher complexity in the seroprevalence curves augmented the uncertainty associated with the posterior distributions of the time in which that reduction in SCR actually occurred and of the ratio between current and past SCR (Figures 4(b) and 4(c)). Adjusting for putative migration effects, the posterior median and mean for the changing point are around 24.5 and 26 years before sampling, two estimates close to the previous ones assuming no migration (around 28 years; Table 3). For the reduction in SCR itself, the respective posterior mean and medians are 75.8% and 75.9%, two estimates slightly more conservative than those obtained for the RCM with no migration effects (93.5% and 95.3%, resp.). Finally, notwithstanding the limited sample size, it was possible to borrow information from the sample in order to update the prior distributions of the residence time of each individual (Figure 4(d)). Many individuals could be assumed as locally born in Jacareacanga because the respective posterior median for the fraction of time living in area was close to 100% (Figure 4(d)). In the remaining cases, there was evidence for the presence of migrants in the sample. In conclusion, although model complexity increased uncertainty of the subsequent parameter estimation, the results provided a more realistic snapshot of the* P. falciparum* malaria history of Jacareacanga.

### 2.5. Detecting Individual Level Heterogeneity in Malaria Exposure

All above models for seropositivity data provide a broad description of the SCR at the population level. Their utility is then limited if one aims to understand more granular, individual level heterogeneities inherent to malaria transmission. For example, a recent study from Cambodia has highlighted the role of age, ethnicity, village of residence, or forest work on the seroconversion of each individual during rainy season [45]. Other examples are the effect of elevation in SCR in northeast Tanzania [12, 13] or the impact of malaria control interventions in western Kenyan highlands [22]. Although age is an intrinsic variable of the above RCM and SIM, the effect of other types of covariates affecting seropositivity suggests adopting a regression-type approach to tackle putative individual level heterogeneity in SCR. This can be easily done by considering the following log-linear model for the SCR of the *i*th individual with a set of *p* covariates *x*_1,*i*_, *x*_2,*i*_,…, *x*_*p*,*i*_:

(17) $$\begin{array}{c} \log\lambda_{i}=\beta_{0}+\beta_{1}x_{1,i}+\beta_{2}x_{2,i}+\cdots +\beta_{p}x_{p,i}, \end{array}$$

where *β*_0_ is the overall effect in absence of covariates and *β*_1_,…, *β*_*p*_ are the regression coefficients associated with each covariate. This regression model is then coupled with RCM or SIM (see (6) and (7)) with stable transmission intensity but describing *λ* with the above model. In the unrealistic situation that malaria infections induce lifelong immunity (see (8)), the inclusion of covariates is facilitated because the resulting model is integrated in the well-known generalized linear model framework via a complementary log-log model for binary variables [47].

Since the analysis must take into account the data from each individual, previous Binomial-product for the sampling distribution (see (9)) is now reconverted into a Bernoulli-product, one Bernoulli distribution per individual; that is,

(18) $$\begin{array}{c} f\left(\;\left\{\;y_{i}\;\right\}\;|\;\left\{\;\pi_{i}\;\right\}\;\right)=\overset{n}{\underset{i=1}{\prod }}\pi_{i}^{y_{i}}{\left(\;1-\pi_{i}\;\right)}^{1-y_{i}}, \end{array}$$

where *y*_*i*_ is the serological status of the *i*th individual, *π*_*i*_ is the probability of the *i*th individual being seropositive, and *n* is the sample size.

In theory, maximum likelihood and Bayesian methods can be applied to estimate all unknown parameters of the above model. In practice, computationally efficient maximum likelihood methods still need to be developed and, therefore, Bayesian methods via MCMC are the most pragmatic approach to data analysis. In absence of prior information about the regression parameters, the usual choice for the respective a priori distribution is to use Gaussian distribution with mean 0 and a sufficiently large standard deviation (e.g., 100). If any prior information exists for the regression parameters, one can alternatively use any eliciting method for Bayesian regression analysis as described elsewhere [48, 49]. 

**Figure 5:**
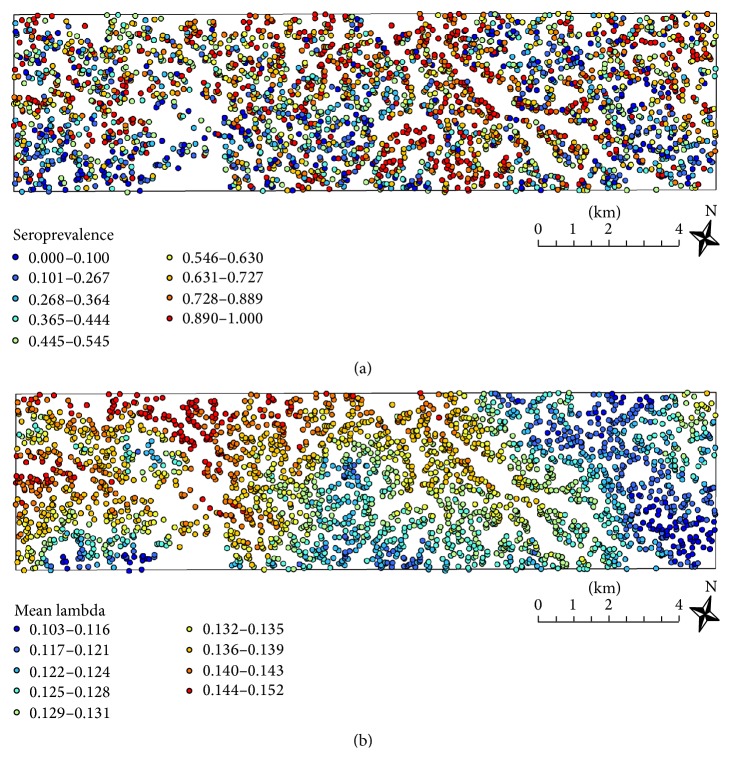
Maps of the western Kenyan highlands showing the distribution of the surveyed households and household level exposure. (a) Map based on the combined seroprevalence for AMA1 and MSP1 antigens. (b) Map based on the posterior mean of SCR adjusting for variations in elevation and gender and use of mosquito control interventions. Each household is represented by a circle and the shading shows the intensity of malaria exposure from blue (low) to red (high).

*Example III* (Rachuonyo South, Kenya). The western Kenyan highlands are currently characterized by low-level endemic and highly heterogeneous* P. falciparum* malaria transmission. To ensure high resolution to detect heterogeneity in malaria exposure, approximately one-third of the total population, around 17,500 individuals, were sampled from a 100 km^2^ area in Rachuonyo South in the western Kenyan highlands [22]. The analysis was focused on* P. falciparum* seropositivity data from about 13,000 individuals with complete data. Combined seropositivity for AMA1 and MSP1 antigens was calculated using the two-component Gaussian mixture model approach for determining seropositivity to each antigen. RCM with stable malaria transmission was then fitted to the data using maximum likelihood methods. The overall SCR was estimated at 0.132, suggesting an overall seroprevalence of 55.2%. When the observed seropositivity of each individual was aggregated to the household level and plotted on a map, there is a substantial variation within the study area (Figure 5(a)). However, the large amount of variation renders it difficult to delineate hotspots of seroprevalence. With this in mind, the previous analysis was then refined in order to take into account available information on gender, elevation, residing or not in a house that received indoor residual spraying in the previous 12 months, and sleeping or not under a bednet the previous night. Similar to the Jacareacanga example, Bayesian methods were applied to the data using noninformative prior distributions for the regression coefficients and SRR. Since a two-component Gaussian mixture model was used for determining seropositivity to each antigen, this extended analysis focused on the RCM model given by (6) where SCR was described by a log-linear regression model including the above-mentioned covariates. Posterior estimates highlighted a significant role of elevation on SCR while the remaining covariates, despite explaining some individual variation in seropositivity, were not statistically significant in the regression model (results not shown) but were maintained due to their known impact on malaria. A new map based on the posterior means of SCR for each individual aggregated to the household level was then generated (Figure 5(b)). This map suggested that significant variation in SCR exists within this 100 km^2^ study area and identifies households with high SCRs. The identification of these putative hotspots of exposure may be instrumental to design future interventions in the study area.

### 2.6. Antibody Acquisition Models

In all models described above, the information on antibody titres is reduced to the proportions of seropositive and seronegative. Alternatively one can analyze data of antibody titres themselves using the antibody acquisition models [50, 51]. In these models, one assumes that the rate at which antibody levels are acquired can be used as a marker for transmission intensity. If an individual's antibody level *A* is boosted at rate *α*(*t*) and decays at rate *r* then antibody levels can be described by the following ordinary differential equation [51]:

(19) $$\begin{array}{c} \frac{dA}{dt}=\alpha \left(\;t\;\right)-rA. \end{array}$$

When malaria transmission is constant over time, the same is assumed for the rate of generation of antibodies in response to infection; that is, *α*(*t*) = *α*. Under the initial condition of *A*(0) = 0, the above equation can be solved analytically to give

(20) $$\begin{array}{c} A\left(\;t\;\right)=\frac{\alpha }{r}\left(\;1-e^{-rt}\;\right), \end{array}$$

where *t* is again regarded as the age of an individual at data collection. The above model can be also extended to include the effect of maternal antibodies [51].

Likewise for seropositivity-based models, historical changes in malaria transmission intensity can also be accounted for. For example, if there was an abrupt reduction in transmission *τ* years before data collection such that the rate of acquisition of antibodies changed from *α*_1_ to *α*_2_, then the expected antibody titre of an individual with age *t* is

(21) At=α2r1−e−rt,t≤τ,α1r1−e−rt−τe−rt+α2r1−e−rτ,t>τ.

The above equation is explained as follows. For individuals born after the change in transmission (*t* ≤ *τ*), the expected antibody dynamics follow exactly as in the constant transmission scenario but with boosting rate *α*_2_ (see (20)). For the individuals born before the change in transmission, one can partition the antibody levels into two terms, the first one referring to the expected antibody levels produced until the change point with boosting rate *α*_1_ discounted by an exponential decay with rate *r* until present time and the second one referring simply to the antibody counts expected to be produced since the change point.

Equations (20) and (21) provide expressions for an individual's antibody titre as a function of age. However, in a population of individuals there is likely to be substantial variation in antibody titres. As mentioned earlier for seropositivity determination, antibody titre data are often approximately Gaussian distributed on a log scale. Therefore, when constructing the sampling distribution for the comparison of the antibody acquisition model with data, one can assume that at age *t* antibody data is log-Normally distributed with parameters *μ* = log⁡(*A*(*t*)) and *σ*. Note that in this interpretation *A*(*t*) is the geometric mean titre (GMT) at age *t* (corresponding to the mean on a log scale). For a random sample of *n* individuals, the respective sampling distribution is given by

(22) $$\begin{array}{c} f\left(\;\left\{\;x_{i}\;\right\}\;|\;\theta ,n\;\right)=\overset{n}{\underset{i=1}{\prod }}\frac{1}{x_{i}\sigma \sqrt{2\pi }}e^{-{\left(\log x_{i}-\log A\left(t_{i}\right)\right)}^{2}/2\sigma^{2}}, \end{array}$$

where *x*_*i*_ and *t*_*i*_ are the antibody titres and age of the *i*th individual, respectively, and *θ* is the parameter vector associated with antibody acquisition model under analysis. This parameter vector is given by *θ* = (*α*, *r*, *σ*) or *θ* = (*α*_1_, *α*_2_, *τ*, *r*, *σ*) if fitting the model with constant malaria transmission intensity or fitting the model with an abrupt reduction in transmission, respectively. Since the above models are nonlinear, parameter estimation can be performed by nonlinear least squares available for the R software or by Bayesian methods via MCMC. For cases where the data are not well described by a log-Normal distribution, alternative sampling distributions will need to be constructed. For example, if a proportion of the population has never been exposed to malaria (i.e., their antibody titres are just background responses), then a zero-inflated log-Normal distribution could be used as an alternative sampling model. Statistical methods to fit that distribution to data can be found elsewhere [52–54]. 

**Figure 6:**
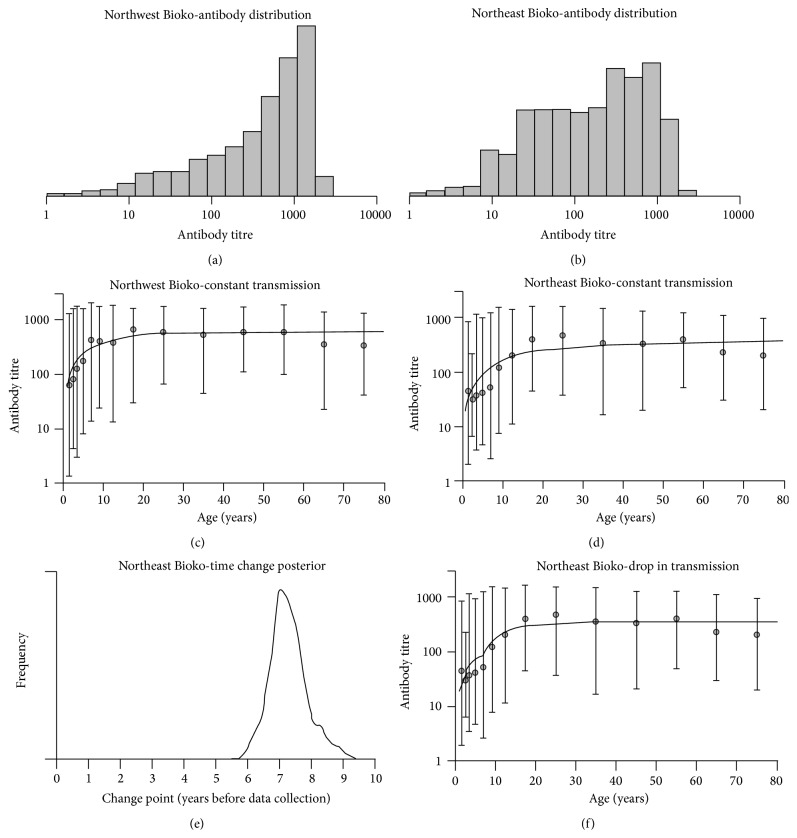
Data analysis of anti-AMA1 antibody titres from Bioko Island using the antibody acquisition models. (a) Sample distribution of antibody titres from northwest region. (b) Sample distribution of antibody titres from northeast region. (c) Antibody acquisition model with constant transmission applied to data from northwest Bioko. (d) Antibody acquisition model with constant transmission applied to data from northeast Bioko. (e) Posterior probability distribution of change point predicted by the antibody acquisition model applied to data from northeast region. (f) Antibody acquisition model with a drop transmission applied to data from northeast region.

*Example I* (Bioko Island continued). To extend previous analysis based on seropositivity data, antibody titre data from northeast and northwest regions of Bioko Island were alternatively analyzed using the above antibody acquisition models. The respective data is shown in Figures 6(a) and 6(b). To compare with previous results, the above antibody acquisition models assuming a constant malaria transmission intensity (Figures 6(c) and 6(d)) and an abrupt reduction in malaria transmission (Figures 6(e) and 6(f)) were fitted to each data set separately. Again, there was evidence for a constant malaria transmission intensity in the northwest region of the island (Figure 6(c)) with an average increase of antibody titres of around 60.4 units per year of exposure (Table 4). In contrast, the antibody acquisition model with constant transmission intensity showed some fitting deficiencies at younger ages for the northeast region (Figure 6(d)), which were eliminated by assuming a drop in malaria transmission intensity (Figure 6(f)). That drop in malaria transmission intensity seemed to have occurred 7 years before sampling, an estimate consistent with the one obtained from seroprevalence data (6 years before sampling; Table 2). According to posterior estimates in Table 4, the average value of antibody acquisition per year decreased from 128 to 20. These estimates suggested a reduction of 84% in malaria transmission intensity, which is in close agreement with a reduction in SCR of 89% estimated from the superinfection model (Table 2).

## 3. Envisioning the Future: Serology and Malaria Elimination

Malaria eradication and elimination are currently in the agenda of various countries worldwide, such as Sri Lanka [3] or Haiti [4]. With this mind, an important question naturally arises: How can one declare if elimination or eradication was actually achieved? Again, serology can help answering this question due to its capacity to detect recent malaria exposure in an apparently asymptomatic population.

As discussed above, the first step of serology data analysis is typically to determine seropositivity from titre data. However, in a malaria elimination setting, seroprevalence is supposedly low in the population, thus, making it difficult to discriminate whether the data comes from a single Gaussian distribution or from a Gaussian mixture model. In this context, the presence of a single Gaussian distribution in the data can be interpreted as indicative of a seronegative population only, thus, suggesting malaria elimination (or eradication). On the other hand, the detection of a mixture distribution indicates the presence of seropositive individuals that might be on their way to seronegativity but also might have been exposed to malaria after a putative elimination event. Hence, sample size determination before data collection ensures to some extent the accuracy of the findings. However, sample size determination is theoretically challenging in the context of mixture distributions because standard asymptotic theory for hypothesis testing does not hold. This implies using less-known statistical methods, such as the bootstrap approach proposed by McLachlan [55] or the adjusted likelihood ratio test derived by Lo et al. [56]. Limited sample size guidelines exist for these alternative methods and, therefore, future research is needed to better help designing malaria surveillance based on serology data.

**Figure 7:**
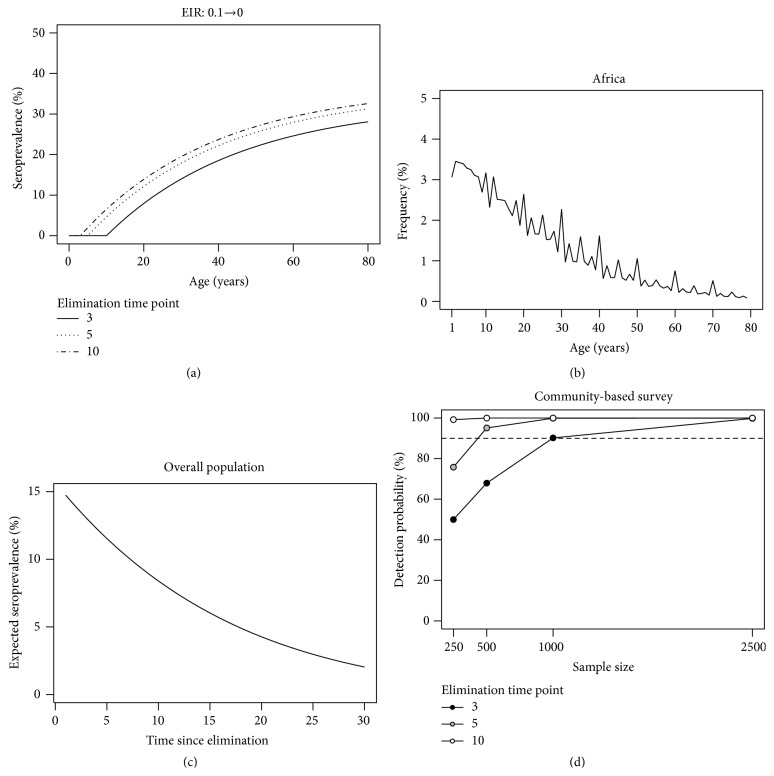
Certifying malaria elimination under a serology-based approach. (a) Expected seroprevalence curve from RCM assuming different elimination time points in relation to data collection. (b) Typical age distribution of an African population. (c) Expected seroprevalence in a random sample taken from a typical African population. (d) Probability to detect elimination as a function of sample size under the assumption of a community-based survey conducted in a typical African population.

Under the assumption of detecting a mixture distribution in the antibody titre data, it was shown above how to infer different sources of heterogeneity in malaria transmission intensity as long as the correct antibodies are analyzed. In this regard, recent research produced a list of putative antigens that can be used in malaria elimination studies for being highly informative on the time to most recent infection [20]. Although malaria elimination and eradication are more likely to be achieved by a slow decrease of malaria transmission intensity towards 0, a reasonable analytical starting point is to aim at using the above RCM and SIM under the assumption of an abrupt reduction in SCR at a given time point before sampling collection. In a malaria elimination setting, current SCR of these models is set at 0 while past SCR should be estimated from the data. The corresponding age-adjusted seroprevalence curve shows a distinctive pattern where children born after the time of malaria elimination are all seronegative while the remainder might or not be seropositive depending on the past malaria exposure before malaria elimination (Figure 7(a)). These latter individuals would slowly revert to a seronegative state over time. For certification purposes, these models must be compared to the ones assuming a (very low) stable malaria transmission intensity for the same data. Therefore, one needs to understand whether data has enough statistical power for detecting disease elimination. To overcome eventual problems later in a study, it is then recommended to perform sample size calculation before collecting data. This problem has recently been tackled for estimating the SCR in stable malaria transmission intensity settings [34]. General guidelines for sample size determination are difficult to put forward because estimation precision and statistical power are intimately related to the age distribution associated with a given study design. In this regard, African studies are more facilitated than those conducted elsewhere because the age distribution tends to be consistent across populations with a decreasing trend from newborns to elders (Figure 7(b)). If such distribution is combined with the expected age-adjusted seroprevalence, one can have an idea of the evolution of overall seroprevalence over time (Figure 7(c)). Since age distribution can vary from one study to another, the minimum sample size for detecting malaria elimination is better calculated by means of data simulation using the expected age distribution for the sample together with the most likely age-adjusted seroprevalence curve for the malaria elimination. As an example, Figure 7(d) shows the power to detect malaria elimination as a function of sample size under the assumption of simple random sampling from a typical African population where the past SCR was conceptually equivalent to 0.1 infectious bites per person per year. As expected, the required sample size decreases with time of the malaria elimination event. For a 90% power, detecting malaria elimination occurring 3, 5, and 10 years before data collection requires sample sizes of 1,000, 500, and <250 individuals, respectively. It is worth noting that the choice of a particular sample strategy involves weighting ethical issues, availability of human and economic resources, and so forth, in order to be officially approved and feasible in real time. Moreover, targeting or oversampling specific age groups might be alternative sampling strategies to reduce sample size. This and other issues will be tackled in a future research.

## 4. Conclusion

In conclusion, serology data in conjunction with mathematical modelling provides a powerful approach to inform epidemiologists on malaria transmission intensity and its putative changes over time. However, serology-based analysis needs to be complemented with any additional data available that would provide an external validation to the serological findings. Imagining the best model for a given data set assumes a constant malaria transmission intensity over time. Checking the official statistics of the malaria cases if available might shed some light on whether such assumption holds true in reality. Similar rationale can be applied to situations where a drop in malaria transmission intensity was detected on the data. This approach of using official data to consolidate serological finding was indeed followed by the study in the Brazilian Amazonia region here analyzed [7]. In this study, the seroconversion rates for* P. vivax* antigens from several sites with different malaria endemicity levels were highly correlated with the corresponding annual parasite indexes compiled by the Brazilian health authorities, suggesting that the serological analysis was capturing the epidemiology of the study sites [7]. In the same line of thought, another study found that seroconversion rates were highly correlated with the parasite rates in northeast Tanzania [13]. Notwithstanding this good agreement between serological findings and other data, it is worth mentioning that the mathematical models for serology data are a simplified abstraction of the real world; a discussion about how robust these models are in practice can be found elsewhere [12]. However, in the words of the statistician George Box [57], all models are wrong but some are useful and that seems to be the case for the mathematical models here presented.

Two main limitations can be pinpointed to the use of a serological approach to malaria epidemiology. The most obvious one is related to which antigens are epidemiologically informative. With this respect, the two antigens most used in practice are MSP1 and AMA1 due to their immunogenicity together with the existence of optimised experimental protocols. Research efforts are currently carried out in order to identify the panel of antigens that would provide the best characterization of the epidemiological status quo of a population [20, 58]. However, these new identified antigens remained to be fully tested in subsequent field studies. One less obvious limitation is the putative lack of statistical power to estimate the seroreversion rate and its putative changes throughout life. This is very clear in low transmission settings where only a few seronegative individuals might result from seroreversion events. Ideally, seroreversion rate is a quantity that is best estimated via longitudinal studies. However, in practice, seroreversion rate is estimated indirectly via cross-sectional data, possibly leading to poor estimation precision as discussed in depth elsewhere [34]. Possible solutions for this problem include using prior information in the analysis or analyzing data from different populations together in order to borrow information from samples where the estimation of seroreversion rate is facilitated. Several future challenges were identified in particular in the context of malaria elimination and eradication. It is worth noting that a serology approach should not be seen as strict to malaria epidemiology with the potential of being applicable to other infectious diseases as long as these are capable of triggering an antibody-mediated immune response in the host. In particular, antibody data is particularly useful to track down the transmission intensity of some neglected tropical diseases, such as Trachoma [39], Chagas [40, 43], or Dengue [57, 59], due to their low endemicity and the lack of clinical symptoms of most infections. However, this requires a deeper knowledge of the antibodies with the highest potential of informing the underlying disease transmission intensity. An interesting idea with public health potential is to use a panel of multidisease antibodies that can be instrumental to know what the infectious agents are in circulation in a given population and their putative dynamics. This idea has not been tested in practice but definitely will require extending the above mathematical models to fully account for the immunological interaction between different diseases.

## Figures and Tables

**Table 1:** Gaussian mixture modelling analyses for determining seropositivity to AMA1 titre data in a sample of around 6400 individuals from Bioko Island using 90% as the cut-off value for the correct classification probability.

Number of components	AIC^a^	Mean (SD)^b^	Definition of	Cut-off values^c^		Classification probabilities^d^		
			*S* ^+^ and *S* ^−^	*c* ^−^	*c* ^+^	*P*_*S*^−^_	*P*_ind_	*P*_*S*^+^_
2	84601.2	59.3 (48.4)	*S* ^−^ = 1, *S* ^+^ = 2	95.9	202.9	31.2	12.7	56.1
		668.1 (450.4)						
3	83395.2	35.8 (26.8)	*S* ^−^ = 1, *S* ^+^ = 2,3	44.6	109.8	19.3	13.8	66.8
		214.0 (115.4)	*S* ^−^ = 1,2, *S* ^+^ = 3	103.8	515.2	32.3	33.3	34.4
		848.3 (425.6)						
4	82887.4	14.1 (9.1)	*S* ^−^ = 1, *S* ^+^ = 2, 3, 4	NA	37.2	—	17.0	83.0
		64.7 (32.4)	*S* ^−^ = 1,2, *S* ^+^ = 3, 4	34.0	149.5	16.1	22.1	61.9
		252.2 (120.6)	*S* ^−^ = 1,2, 3, *S* ^+^ = 4	135.4	560.3	36.4	31.3	32.3
		873.2 (420.6)						

^a^The best model is the one providing the lowest estimated value.

^b^Mean and standard deviation (SD) are for each Gaussian component in the model ordered by the corresponding average titres.

^c^ *c* ^−^ and *c* ^+^ are the cut-off values for determining the seronegative and seropositive populations, respectively.

^d^ *P*_*S*^−^_, *P*_ind_, and *P*_*S*^+^_ are the estimated classification probabilities of seronegative, indeterminate, and seropositive individuals, respectively.

**Table 2:** Maximum likelihood estimates for seroconversion and seroreversion rates (SCRs and SRRs, resp.) of antibodies against AMA1 expected for northwest and northeast regions of Bioko Island using the reversible catalytic and superinfection models (RCMs and SIM, resp.) under the assumptions of constant malaria transmission intensity over time and an abrupt reduction in malaria transmission at a given change time point before data collection.

Region	Model	Malaria transmission	SCR (95% CI)	SRR (95% CI)	log-likelihood
Northwest	RCM	Constant	0.286 (0.249, 0.328)	0.008 (0.005, 0.015)	−71.31
	SIM	Constant	0.359 (0.307, 0.419)	0.091 (0.069, 0.120)	−69.71
Northeast	RCM	Constant	0.124 (0.109, 0.141)	0.006 (0.004, 0.011)	−96.87
	SIM	Constant	0.139 (0.119, 0.163)	0.039 (0.028, 0.056)	−102.95
	RCM	Abrupt reduction (change point = 6)	0.274 (0.200, 0.376)	0.009 (0.005, 0.014)	−84.25
			0.077 (0.058, 0.100)		
	SIM	Abrupt reduction (change point = 6)	0.900 (0.431, 1.879)	0.150 (0.097, 0.232)	−83.37
			0.098 (0.075, 0.129)		

**Table 3:** Bayesian analysis of *P. falciparum* seropositivity data from Jacearecanga where RCM_red_, RCM_red+mig_, and RCM_behavior_ denote the reversible catalytic models assuming an abrupt reduction in SCR only, an abrupt reduction together with migration effects, and a change in SCR due to a behavioral factor dependent on a given age cut-off.

Model	Parameter	Posterior estimates		
		Mean	Median	95% credible interval^a^
RCM_red_	Past SCR	0.436	0.386	0.099–0.948
	Current SCR	0.019	0.019	0.009–0.033
	Time elapsed since reduction	27.6	28.0	22.0–33.0
RCM_red+mig_	Past SCR	0.292	0.192	0.052–0.916
	Current SCR	0.038	0.037	0.013–0.067
	Time elapsed since reduction	24.5	26.0	4.0–39.0
RCM_behavior_	Baseline SCR	0.051	0.046	0.019–0.106
	Risk SCR	0.654	0.693	0.153–0.988
	Age cut-off	28.9	29.0	26.0–33.0

^a^Credible interval based on 2.5% and 97.5% quantiles of the respective posterior distribution.

**Table 4:** Parameter estimates for antibody acquisition models applied to anti-AMA1 antibody titre data (AU: arbitrary units) from northwest and northeast region of Bioko Island. Estimates are presented as posterior medians with 95% credible intervals in brackets.

Region	Malaria transmission	α_1_	α_2_	τ	*r*	σ
Northwest	Constant	60.4 (50, 65)	—	—	0.098 (0.07, 0.12)	1.36 (1.32, 1.41)
Northeast	Constant	20.2 (18, 23)	—	—	0.053 (0.04, 0.07)	1.36 (1.31, 1.42)
	Drop	128 (65, 232)	20 (17, 23)	7.2 (6.2, 8.7)	0.16 (0.11, 0.21)	1.33 (1.28, 1.39)
